# Chromogranin A as a Novel Biomarker of Irritable Bowel Syndrome in Adults: A Systematic Review and Meta-Analysis

**DOI:** 10.34172/mejdd.2025.418

**Published:** 2025-04-30

**Authors:** Virly Nanda Muzellina, Nicolas Daniel Widjanarko, Jonathan Christianto Subagya, Steven Alvianto

**Affiliations:** ^1^Department of Internal Medicine, Faculty of Medicine, Universitas Indonesia, Central Jakarta, Indonesia; ^2^Department of General Medicine, Faculty of Medicine and Health Sciences, Atma Jaya Catholic University of Indonesia, North Jakarta, Indonesia

**Keywords:** Irritable bowel syndrome, Chromogranin A, Gaster, Duodenum, Colon

## Abstract

**Background:**

Irritable bowel syndrome (IBS) is a chronic condition characterized by recurring abdominal discomfort and irregular bowel movements. Currently, IBS diagnosis lacks specific radiological, biochemical, or endoscopic markers. Chromogranin A (CgA), a gastrointestinal protein, shows variation between patients with IBS and healthy controls. This study aimed to evaluate differences in CgA concentrations between these groups.

**Methods:**

This review was conducted in 2023 using the PRISMA 2020 guidelines. All observational research was retrieved from MEDLINE, EBSCO-Host, ScienceDirect, and ProQuest electronic databases using a predefined search strategy. Study quality was assessed using the Newcastle-Ottawa Scale (NOS), and meta-analysis was conducted using Review Manager (RevMan) 5.4.

**Results:**

Nine out of 14 studies eligible for meta-analysis revealed significantly higher CgA cell density (*P*=0.0001) in all patients with IBS compared with controls. This difference persisted across colon regions (left: *P*=0.04, right: *P*=0.0009) and duodenum (*P*<0.00001). Subgroup analysis found no significant disparity in CgA cell density between diarrhea and constipation-predominant IBS within the duodenum or colon.

**Conclusion:**

CgA cell density showed trends toward IBS compared with control groups, with significant concentration differences found in the duodenum, left, and right colon. Therefore, current findings might offer a histopathological approach to confirm the IBS diagnosis.

## Introduction

 Irritable bowel syndrome (IBS) is a persistent and incapacitating disorder involving the interaction between the gut and the brain, identified by recurrent abdominal pain and irregular bowel movements.^1^ Although considered functional, it significantly disrupts affected individuals’ normal life and work due to its function alterations.^2^ Diagnosis of IBS currently lacks specific radiological, biochemical, or endoscopic markers. Therefore, the primary means of identifying IBS relies on a comprehensive clinical evaluation, focusing on the classical symptom triad of abdominal discomfort/pain, altered bowel habits, and bloating/abdominal distension. IBS is further categorized into four subtypes determined by the predominant stool pattern reported by the individual, namely IBS with constipation (IBS-C), IBS with diarrhea (IBS-D), IBS with mixed bowel habits (IBS-M), or IBS unclassified (IBS-U).^3^

 In a study conducted by Sperber and colleagues, the global prevalence of IBS was 8.8%. Regional variations were observed, with the highest prevalence reported in Latin America at 17.5% and the lowest in the Middle East/Africa at 5.8%. Additionally, the study noted that over 50% of patients with IBS were women, with rates in women being 1.5 to 3 times higher than in men. Asia ranks as the second-highest region regarding IBS prevalence, affecting approximately 9.6% of the population.^4^ Research also indicates that 24.3% of patients with IBS were absent from work, leading to disruptions in work productivity, as reported by 86.8% of these patients. This highlights the significant impact that IBS can have on both work attendance and productivity, necessitating early diagnosis and prompt treatment.^5^

 Chromogranin A (CgA), a 49 kDa protein consisting of 431-445 amino acid residues, was first isolated from the adrenal medulla’s chromaffin cells. CgA is one of the three classic granins (a unique acidic and soluble secretory protein), besides Chromogranin B (CgB) and Chromogranin C (CgC)/Secretogranin II.^6^ In the gastrointestinal tract (GI), CgA has been considered a common biomarker for gut endocrine cells.^7^ Emerging evidence revealed that the changes in CgA cell density played a major role in contributing to the pathogenesis of IBS, as there were aberrants in CgA cell density in patients with IBS.^6^ It has been demonstrated that dietary recommendations, especially low-fermentable oligosaccharides, disaccharides, monosaccharides, and polyols (FODMAP) diets, reduce IBS symptoms and restore normal CgA cell densities in the GI tract. Improvement in symptoms is correlated with this normalization, suggesting that CgA may play a part in the pathogenesis of IBS.^8^

 Patients with IBS have normal, decreased, or raised serum CgA levels.^9^ CgA concentration experienced reduction in duodenum,^6^ ileum,^7^ colon,^10^ and gastric,^11^ along with no changes observed in the rectum.^12^ Sidhu and others identified elevated blood CgA levels in a subset of patients with diarrhea-predominant IBS (IBS-D), suggesting that this increase could be attributed to enterochromaffin cell hyperplasia, particularly in post-infectious IBS cases.^13^ In contrast, El-Salhy and colleagues considered changes in serum CgA levels to be clinically insignificant and found no significant differences compared with healthy controls. Instead, they observed a reduced density of CgA-containing cells.^9^ Furthermore, the study by Mujagic and others showed an increase in CgA density in fecal samples of patients with IBS as compared with controls (23.3 ± 28.5 versus 14.6 ± 15.7, respectively, *P* = 0.001),^14^ which represented the colonic CgA concentration. These lack of consistent findings and conflicting results from different studies cast doubt on this issue, necessitating further meta-analysis to solve this.

 Each region of the GI tract boasts a unique cast of hormone-producing cells, reflecting their individual tasks in processing and absorbing nutrients. However, in IBS, this normally balanced structure of cell function can be disrupted by perturbations in the gut microbiome and chronic low-grade inflammation, potentially impacting the concentration of CgA cells.^15^ Based on the hypothesis that altered CgA cell density may represent a novel feature of IBS, this study aimed to evaluate and summarize the differences in CgA concentration between individuals with IBS and healthy controls. Our review represents the first exploration of the unique endocrine cell profile in IBS, potentially opening new avenues for understanding the course of the disease.

## Materials and Methods

 The Preferred Reporting Items for Systematic Reviews and Meta-Analysis (PRISMA) 2020 statement was used as a guideline to design and conduct this systematic review and meta-analysis.^16^ The protocol was registered at the International Prospective Register of Systematic Reviews (PROSPERO) on December 13^th^ 2023, with registration number CRD42023488983.

###  Eligibility Criteria

####  Type of Studies

 This systematic review included all published observational studies examining the difference of CgA cell density between adult patients with IBS and healthy controls in several parts of the GI tract. Conversely, studies falling under the categories of reviews, case reports, case series, conference abstracts, book sections, commentaries/editorials, and papers entailing non-human subjects were excluded. To preclude potential bias arising from the therapeutic intervention altering CgA concentration, we collected and analyzed CgA concentration data from interventional study participants before they received any treatment.

####  Participants

 Participants in this study adhered to strict inclusion and exclusion criteria. Inclusion required being 18 years or older with a primary diagnosis of IBS confirmed by Rome criteria, a smoke-free lifestyle, and no GI medications within the past 48 hours. Individuals excluded were those with: (a) a predominant upper GI functional disorder or relevant systemic disease, (b) a history of specific medication or laxative use, (c) recent antibiotic exposure, (d) need for psychotropic drugs or specific contraceptives, (e) clinical alarm symptoms, (f) pregnancy or breastfeeding, or (g) prior abdominal surgery.

###  Variable and Outcome of Interest

 This study aimed to evaluate the CgA cell density between IBS and healthy subjects in several parts of the GI tract, namely gaster, duodenum, ileum, and colonic parts.

###  Search Strategy and Study Selection

 A literature search was carried out on 5^th^ December 2023 on several electronic databases, including MEDLINE, EBSCO-Host, Science Direct, and ProQuest, to retrieve eligible studies. Four independent authors conducted this step using combinations of the following Medical Subject Heading (MeSH) keywords: *(“Chromogranins” OR “Chromogranin A” OR “secretogranin”) AND (“Irritable Bowel Syndrome” OR “Diarrhea” OR “Constipation”)*. Further detail is described in Supplementary file 1 (Table S1-S5). The PECOTS-SD criteria (participant, exposure, comparator, outcomes, time, setting, and study design) are as follows:

- Patients: Adult patients ( > 18 years) with a primary diagnosis of IBS confirmed by Rome criteria, a smoke-free lifestyle, and no GI medications within the past 48 hours
- Exposure: All types of IBS include IBS-C, IBS-D, or IBS-M
- Comparator: Healthy patients (control)
- Outcomes: CgA cell density
- Time: No publication’s time restriction
- Settings: Participants visiting medical facility
- Study Design: Observational study design

All studies obtained were exported into the Zotero reference manager software and then checked for duplication, followed by titles and abstract screening. All authors performed the assessment independently, and studies were excluded when the title and/or abstract were not appropriate for this review. All authors reviewed the selected papers in full-text assessment using the aforementioned eligibility criteria. The differences observed were settled among all team members.

###  Data Collection Process

 The included studies were analyzed, and the following data were extracted: first author, publication year, country of origin, study design, sample sizes, age and sex percentage of the subjects, population characteristics, inclusion or exclusion criteria of participants, CgA sources and detection method, IBS types, and summary of the findings.

###  Summary Measures

 The concentration of CgA cells was measured and reported in six anatomical segments of the GI tract (gastric antrum and corpus, ileum, duodenum, left and right colon) for both patients with IBS and healthy control subjects. Data presentation employed mean ± standard deviation (SD) for normally distributed data and median (interquartile range) for non-normally distributed data. Additionally, *P* values were provided to indicate statistically significant differences between the groups.

 We converted values from studies that did not report in the form of mean and standard deviation using the formula proposed by Wan et al^17^ and Luo et al.^18^ The aforementioned formula requires data of sample size (N), lower quartile (Q1), middle quartile (Median/Q2), and upper quartile (Q3), which can be extracted from each original study.

###  Assessment of Risk of Bias/Quality Assessment

 The Newcastle-Ottawa Scale (NOS) was applied to evaluate each study, covering cohort, case-control, and cross-sectional designs, with tools consisting of different evaluation aspects. For cohort studies, the domain of assessment included (1) the exposed cohort representativeness, (2) the non-exposed cohort selection, (3) exposure ascertainment, (4) demonstration that the outcome of interest was absent at the study’s outset, (5) comparability, (6) the outcome assessment, (7) the duration of follow-up, and (8) sufficiency of the cohort follow up. Meanwhile, for case-control analyses, the domain of assessments was (1) adequate case definition, (2) the case representativeness, (3) the control selection, (4) the control definition, (5) comparability, (6) the exposure ascertainment, (7) similar ascertainment method for cases as well as controls, and (8) non-response rate. For cross-sectional studies, the domain of assessments was (1) the sample representativeness, (2) the size of samples, (3) non-respondents, (4) the exposure ascertainment, (5) comparability, (6) the outcome assessment, as well as (7) statistical test. Conversion of the final score is qualified as good when there are 3 or 4 stars in the domain related to selection, AND 1 or 2 stars in the domain concerning comparability, AND 2 or 3 stars in the domain related to outcome/exposure; qualified as fair when 2 stars in the domain related to selection, AND 1 or 2 stars in the domain concerning comparability, AND 2 or 3 stars in the domain related to outcome/exposure. Two reviewers independently assessed each article, and any differences were discussed by the review team to reach an agreement.

###  Synthesis of Results and Statistical Analysis

 Review Manager (RevMan; Cochrane Collaboration) version 5.4 was used to extract and pool the data for quantitative synthesis.^19^ For the analysis, all patients were classified into two groups to obtain the difference in CgA cell density between patients with IBS and control groups. Statistical analyses were carried out for between-group comparison using totals and subtotals with 95% CI. Furthermore, subgroup analyses were conducted to investigate potential discrepancies in CgA concentration based on IBS subtypes, specifically constipation-dominant (IBS-C) and diarrhea-dominant (IBS-D).

 Some studies reported primary outcomes using different evaluation or calculation methods; hence, meta-analyses were conducted with a random effects model. This model offered each study an equal weight. Furthermore, it enables extrapolating the pooled results to a wider population in the context of future research endeavors. Combined effect measures for continuous data were calculated using the inverse variance method, with standardized mean differences (SMDs) serving as the most appropriate effect size measure.

 A funnel plots test was carried out to inspect the potential publication bias, in which the difference of each study was plotted by the inverse of its SE. Heterogeneity across trials was assessed using the I^2^ statistic. An I^2^ value less than 25% was considered subtle, 25%-50% showed low, 50% - 75% signified moderate, and more than 75% implied high heterogeneity. A *P* value less than 0.05 was considered significant.

## Results

**Figure 1:**
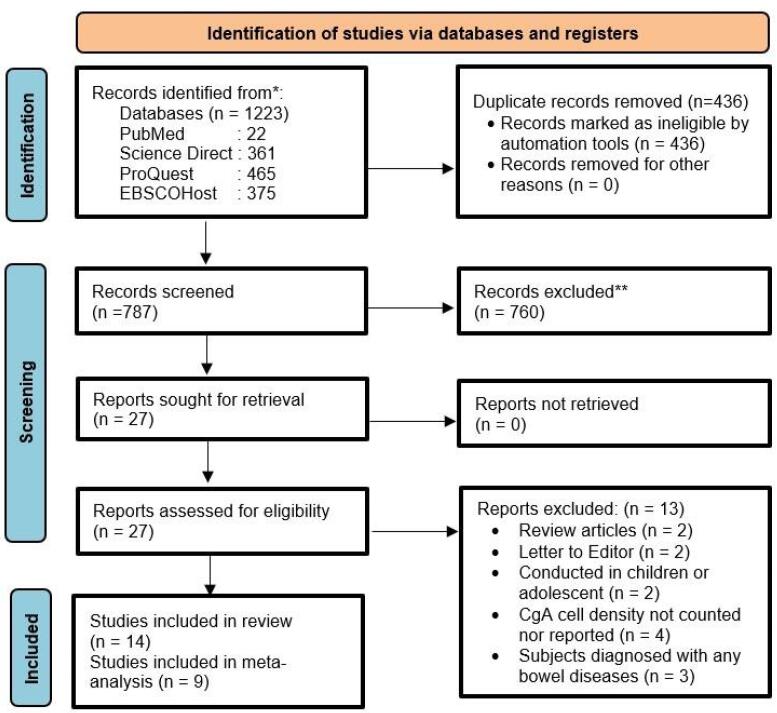
PRISMA 2020 flow diagram of included studies

Figure 1 encapsulates a graphical representation delineating the research selection process and its outcomes. The search strategy produced 1223 potentially pertinent studies. Following the elimination of duplicates, 787 studies met the criteria for title and abstract screening. Following the predefined selection criteria, 27 studies were singled out for comprehensive full-text assessment. Among these, two were review articles, two were letter-to-editor submissions, two were conducted in the pediatric or adolescent population, four failed to report or quantify CgA cell density, and three encompassed patients diagnosed with alternative bowel diseases. Ultimately, 14 studies were incorporated into the systematic review, with nine meeting the eligibility criteria for inclusion in the meta-analysis. No unpublished studies meeting the inclusion criteria were identified, thereby exerting no impact on the overall conclusions drawn from our review.

###  Characteristics of the Included Studies

 A total of 14 studies met the inclusion criteria. The included studies were nine case-control, one cross-sectional, and four cohort studies. Among the 14 studies, nine were conducted in Norway, two in the Netherlands, one in Sweden, one in Croatia, and one in the United Kingdom. Further characteristics of the included studies, including first author, publication year, country of origin, study design, sample sizes, age and sex percentage of the subjects, population characteristics, inclusion and exclusion criteria of participants, chromogranin detection method, chromogranin sources, IBS types, and summary of the results were extracted and reported in Table S6.

 The included IBS participants were 1254, with 935 females and 319 males. The mean age ranged from 32–56 years in IBS participants and 38–54 years in control subjects. Some studies mentioned the participants’ IBS types, namely IBS-D, IBS-C, IBS-M, and IBS-U, while others gave no information on the participants’ IBS types. Furthermore, the chromogranin sources reported varied across studies, with a total of three studies on the duodenum, two studies on the ileum, two studies on the gaster, four studies on the colon, three studies on the rectum, three studies on feces, and two studies on serum.

###  Quality Assessment

 Our exhaustive analysis involved 14 studies, each employing rigorous evaluation tools to scrutinize the risk of bias. All studies underwent assessment using the Newcastle-Ottawa Scale (NOS) based on the study design (case-control, cohort, cross-sectional), as shown in Table 1. Three out of the four case-control studies exhibited good quality, while one had poor quality. Among the eight cohort studies, six demonstrated good quality, whereas two had fair quality. The only cross-sectional study had satisfactory quality.

**Table 1:** Results of quality assessment using Newcastle-Ottawa Scale (NOS) adopted for case-control, cohort, and cross-sectional studies

**Case-Control studies**									
**First author, ** **Year of publication **	**Selection**				**Comparability:** **Cases and controls on the basis of the design or analysis**	**Outcome**			**Overall Risk of Bias**
	**S1:** **Is the case definition adequate?**	**S2:** **Representativeness of the cases**	**S3:** **Selection of controls**	**S4:** **Definition of controls**		**O1:** **Ascertainment of exposure**	**O2:** **Same ascertainment method for cases and controls**	**O3:** **Non-response rate**	
Mazzawi et al,^8^ 2016	*	*	*	*	**	*	*	*	Good quality
Mazzawi et al,^10^ 2015	*	*	*	*	**	*	*	*	Good quality
Mazzawi et al,^11^ 2014	*	*	*	*	**	*	*	*	Good quality
Sidhu et al,^13^ 2009	*	*	*	*		*	*	*	Poor quality
**Cohort studies**									
**First author, ** **Year of publication**	**S1:** **Representativeness of the exposed cohort**	**S2:** **Selection of the non exposed cohort**	**S3:** **Ascertainment of exposure**	**S4:** **Demonstration that outcome of interest was not present at start of study**	**C:** **Comparability of cohorts on the basis of the design or analysis**	**O1:** **Assessment of outcome **	**O2:** **Was follow-up long enough for outcomes to occur**	**O3:** **Adequacy of follow up of cohorts**	**Overall Risk of Bias**
Mujagic et al,^14^ 2016	*	*	*	*	**	*	*	0	Good quality
Mujagic et al,^22^ 2017	*	*	0	*	**	*	*	*	Good quality
Öhman et al,^23^ 2012	*	*	*	*	**	*	*	0	Good quality
El-Salhy et al,^21^ 2017	*	*	*	0	*	*			
El-Salhy et al,^9^ 2010	*	*	*	*	*	*	*	0	Good quality
El-Salhy et al,^12^ 2012	*	*	*	0	*	*	*	0	Fair quality
El-Salhy et al,^7^ 2013	*	*	*	0	*	*	*	0	Fair quality
El-Salhy et al,^20^ 2014a	*	*	*	*	**	*	*	0	Good quality
El-Salhy et al,^6^ 2014b	*	*	*	*	**	*	*	0	Good quality
**Cross-sectional studies**									
**First author, Year of publication**	**S1:** **Representativeness of the sample**	**S2: Sample Size**	**S3:** **Non-respondents**	**S4: ** **Ascertainment of the exposure (risk factor):**	**C: ** **Comparability of subjects in different outcome groups on the basis of design or analysis**	**E1: Assessment of outcome**	**E2: ** **Statistical test**	**Overall Risk of Bias**	
Pletikosic et al,^24^ 2015	*	0	*	*	0	*	*	Satisfactory studies	

###  Final Results

 Nine studies in the quantitative synthesis showed numerous results of chromogranin cell density in patients with IBS. Overall, most of the studies reported significant differences (*P* = 0.0001) in CgA cell density in all types of patients with IBS compared with controls, except for Mazzawi and colleagues ^8^ in ileum (*P* = 0.9819) and El-Salhy et al^12^ in all parts of the colon (*P* = 0.5260). In IBS-C, El-Salhy et al,^9,12^ found non-significant differences of CgA concentrations in IBS-C compared with controls (*P* = 0.0796; *P* = 0.7788, respectively). While in IBS-D compared with controls, El-Salhy et al^9^ found a significant difference in CgA cell density between both groups (*P* = 0.0001), and El-Salhy et al^12^ reported no significant difference (*P* = 0.5109). Further results are shown in Table 2.

**Table 2:** Results for CgA concentrations (cell density) in IBS compared with controls

**First author, Year**	**Gastric antral CgA concentration (cell density)**						
	**All types of IBS**			**Control**			***P*** ** value**
	**Mean **	**SD**	**Participant**	**Mean **	**SD**	**Participant**	
Mazzawi^11^ 2014	28.5	24.32	14	87.7	76.12	14	0.01
El-Salhy^6^ 2014b	372.7	43.6	76	272.5	28.6	59	0.0001
**First author, Year**	**Gastric corpal CgA concentration (cell density)**						
	**All types of IBS**			**Control**			***P*** ** value**
	**Mean **	**SD**	**Participant**	**Mean **	**SD**	**Participant**	
Mazzawi^11^ 2014	62.6	34.79	14	147.9	59.05	14	0.0001
El-Salhy^6^ 2014b	391.3	43.4	76	254.1	24.6	59	0.0001
**First author, Year**	**Ileum CgA concentration (cell density)**						
	**All types of IBS**			**Control**			***P*** ** value**
	**Mean **	**SD**	**Participant**	**Mean **	**SD**	**Participant**	
El-Salhy^7^ 2013	28.6	20.78	98	63.2	22.86	27	0.0001
Mazzawi^8^ 2016	48.4	160.52	11	47.4	31.1	14	0.9819
**First author, Year**	**Left colon CgA concentration (cell density)**						
	**All types of IBS**			**Control**			***P*** ** value**
	**Mean **	**SD**	**Participant**	**Mean **	**SD**	**Participant**	
El-Salhy^9^ 2010	22.7	14.0	41	31.1	15.0	59	0.0057
Mazzawi^10^ 2015	21.9	9.73	13	49.6	22.45	14	0.0004
**First author, Year**	**Right colon CgA concentration (cell density)**						
	**All types of IBS**			**Control**			***P*** ** value**
	**Mean **	**SD**	**Participant**	**Mean **	**SD**	**Participant**	
El-Salhy^9^ 2010	20.5	13.0	41	34.8	14.0	59	0.0001
Mazzawi^10^ 2015	16.7	6.85	13	49.6	22.45	14	0.0001
**First author, Year**	**Duodenal CgA concentration (cell density)**						
	**All types of IBS**			**Control**			***P*** ** value**
	**Mean **	**SD**	**Participant**	**Mean **	**SD**	**Participant**	
El-Salhy^9^ 2010	25.6	22.0	41	50.5	21.0	59	0.0001
El-Salhy^20^ 2014a	89.5	7.2	203	446.1	16.0	86	0.0001
Mazzawi^8^ 2016	36.9	35.2	11	235.9	119.36	14	0.0001
**First author, Year**	**Duodenal CgA concentration (cell density)**						
	**IBS-C**			**Control**			***P*** ** value**
	**Mean **	**SD**	**Participant**	**Mean **	**SD**	**Participant**	
El-Salhy^9^ 2010	29.6	22.0	18	50.5	21.0	59	0.0005
El-Salhy^20^ 2014a	69.8	100.5	76	446.1	16.0	86	0.0001
**First author, Year**	**Duodenal CgA concentration (cell density)**						
	**IBS-D**			**Control**			***P*** ** value**
	**Mean **	**SD**	**Participant**	**Mean **	**SD**	**Participant**	
El-Salhy^9^ 2010	21.4	12.0	23	50.5	21.0	59	0.0001
El-Salhy^20^ 2014a	76.7	9.6	80	446.1	16.0	86	0.0001
**First author, Year**	**All parts of colon CgA concentration (cell density)**						
	**All types of IBS**			**Control**			***P*** ** value**
	**Mean **	**SD**	**Participant**	**Mean **	**SD**	**Participant**	
El-Salhy^9^ 2010	21.3	13.0	41	33.1	14.0	59	0.0001
El-Salhy^12^ 2012	190.2	98.03	47	206.3	115.35	27	0.5260
Mazzawi^10^ 2015	38.6	13.34	13	83.3	37.7	14	0.0004
Mujagic^14^ 2016	23.3	28.5	196	14.6	15.7	160	0.0006
**First author, Year**	**All parts of colon CgA concentration (cell density)**						
	**IBS-C**			**Control**			***P*** ** value**
	**Mean **	**SD**	**Participant**	**Mean **	**SD**	**Participant**	
El-Salhy^9^ 2010	26.4	14.0	18	33.1	14.0	59	0.0796
El-Salhy^12^ 2012	195.3	148.63	19	206.3	115.35	27	0.7788
**First author, Year**	**All parts of colon CgA concentration (cell density)**						
	**IBS-D**			**Control**			***P*** ** value**
	**Mean **	**SD**	**Participant**	**Mean **	**SD**	**Participant**	
El-Salhy^9^ 2010	18.9	12.0	23	33.1	14.0	59	0.0001
El-Salhy^12^ 2012	188.8	77.78	28	206.3	115.35	27	0.5109

###  Meta-Analysis Results

**Figure 2:**
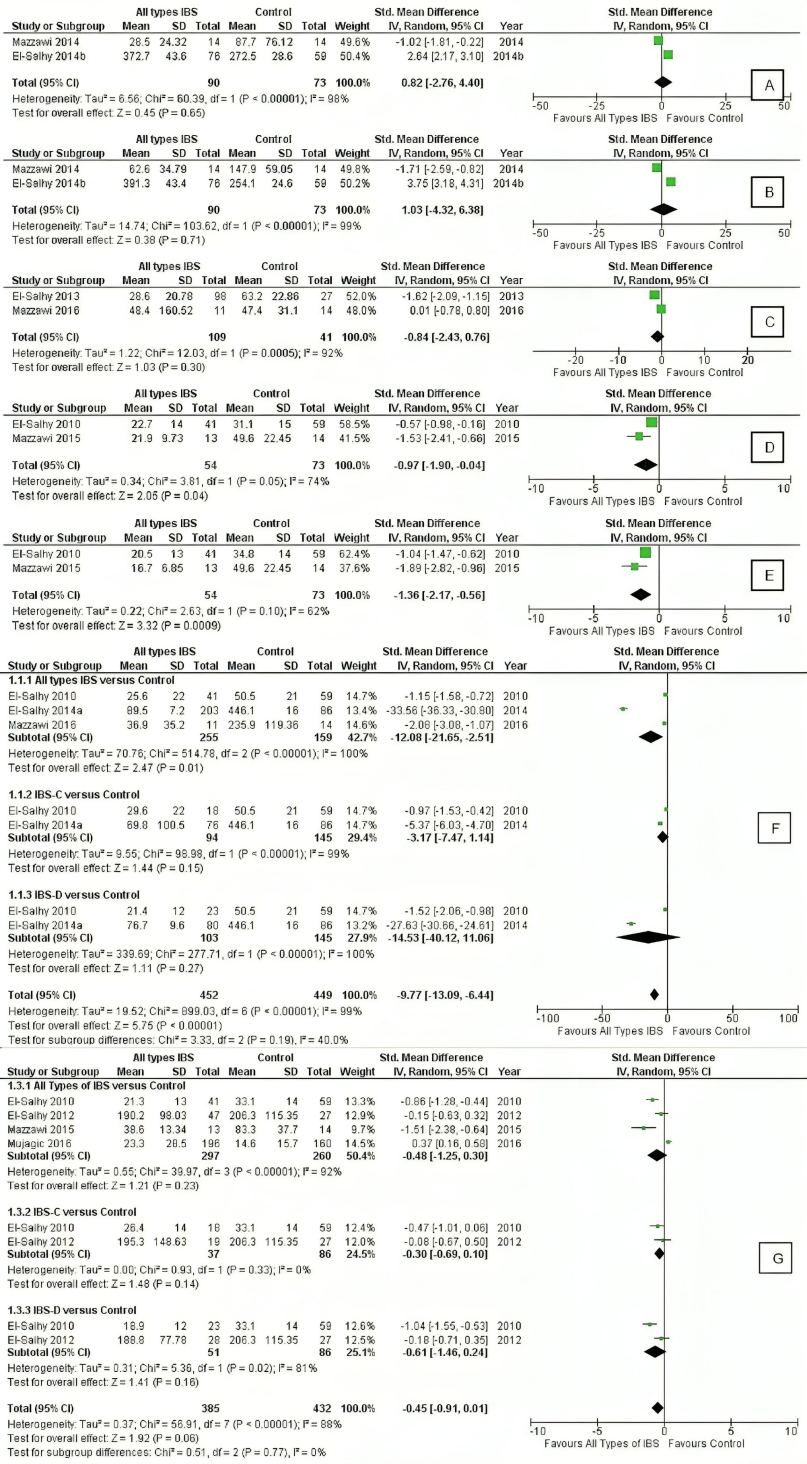
Meta-Analysis Results [Forest Plot] for Chromogranin A Cell Density (Concentration) in: **A**. Gastric Antral; **B**. Gastric Corpal; **C**. Ileum; **D**. Left Colon; **E**. Right Colon; **F**. Duodenum; **G**. All parts of colon. Subgroup analysis for the Duodenum and All parts of the colon based on the types of IBS were shown in Figures **F** and **G**, respectively

**Figure 3:**
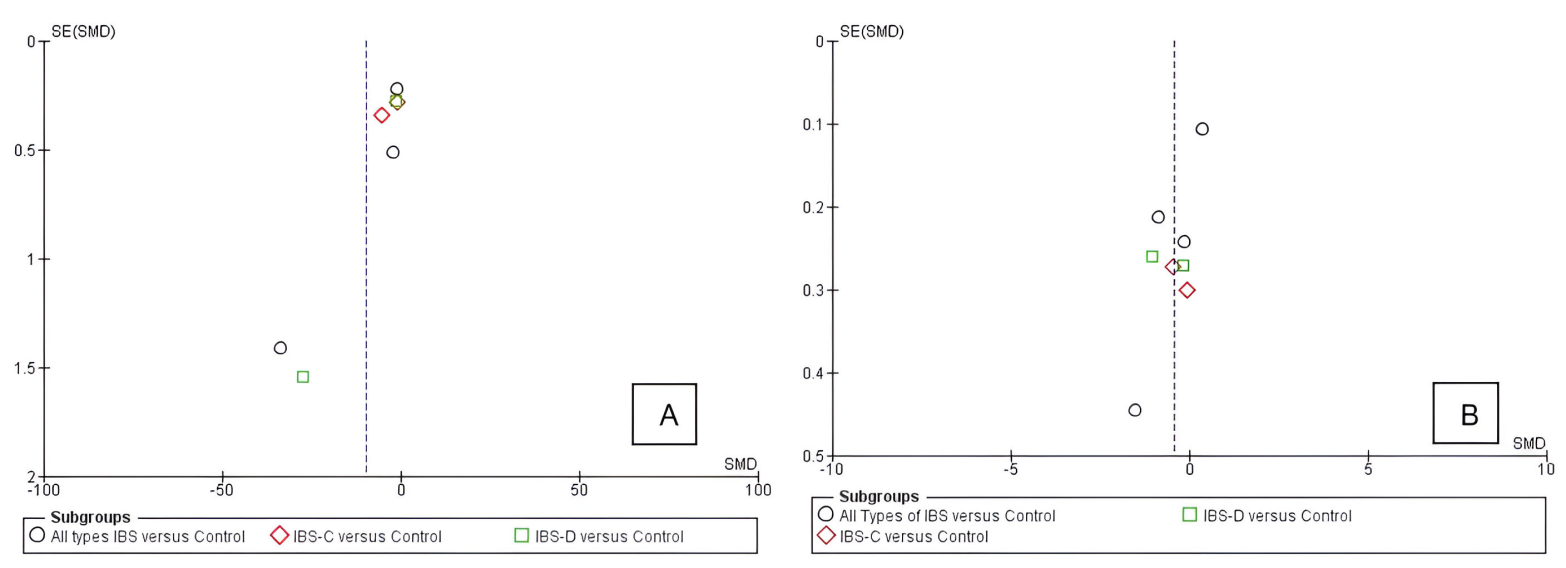
Publication Bias [Funnel Plot] for Chromogranin A Cell Density (Concentration) Subgroup analysis for (**A**) Duodenum and (**B**) All parts of the colon based on the IBS types

 The quantitative synthesis results for CgA concentration in gastric antral, gastric corpal, ileum, left colon, right colon, duodenum, and all parts of the colon are shown in Figure 2. Based on the results, three outcomes (left colon, right colon, and duodenum) showed a significant CgA concentration difference between IBS and control groups (*P* = 0.04, 0.0009, < 0.00001, respectively). The I^2^ test results indicated high heterogeneity in CgA cell density across various GI locations. Values ranging from 88% to 99%, with gastric antrum, corpus, ileum, duodenum, and all colon segments demonstrated substantial variability, while moderate heterogeneity was observed in the left and right colon (74% and 62%, respectively). The subgroup analysis for duodenal and colonic CgA cell density revealed no significant differences across different IBS (IBS-D and IBS-C) types, as shown in Figure 3.

###  Publication Bias Analysis

 Standard error (SE) calculation for all included studies was performed using the formula: SE = (upper CI limit – lower CI limit)/3.92, or by calculating the square root of the calculated error variance (v). Subgroup analysis for the duodenum and all parts of the colon were plotted in separate funnel plots. All individual studies were located on both sides of the vertical line, showing the symmetrical distributions of cumulative effect sizes. The higher the SE, the lower the position of the study in the inverted funnel, showing low power compared with others. Considering the conformity of the funnel plot, publication bias was conceivably low.

## Discussion

###  Gastric CgA Cell Density

 Our result revealed a non-significant difference in gastric CgA cell density between IBS and control groups, both in the antrum (SMD = 0.82, 95% CI: -2.76, 4.40, *P* = 0.65) and corpus parts (SMD = 1.03, 95% CI: -4.32, 6.38, *P* = 0.71). Two studies reported CgA concentration in the stomach showed conflicting findings, with Mazzawi et al^11^ reporting a decrease and El-Salhy^6^ documented an increase of CgA cell density in the stomach, although cumulative results showed control groups exhibited lower levels of CgA.

 Under normal circumstances, the gastric epithelium harbors four distinct endocrine cell populations, distinguished by their hormone production: serotonin (5-HT), somatostatin (SST), ghrelin, and gastrin. SST and 5-HT cells are distributed throughout the corpus and antrum, whereas ghrelin cells are exclusively found in the corpus, and gastrin-producing G cells are restricted to the antral domain.^25^ This could explain the abundant of antral CgA-immunoreactive cells secreting gastrin, 5-HT, and SST, while those in the corpus secrete ghrelin, 5-HT, and SST.^26^

 CgA concentration changes in the stomach varied across IBS subtypes. Both the antrum and corpus regions showed increased CgA-immunoreactive cell density in patients with IBS-C compared with controls. Patients with IBS-M only exhibited decreased density in the antrum.^6^ More specifically, ghrelin-secreting cell density reportedly differs across subtypes, with lower levels in IBS-C and higher levels in IBS-D compared with healthy individuals. Therefore, while patients with IBS-D showed no overall change in CgA cell density across both regions, alterations in specific endocrine cell types within these populations remain a possibility.^6^

###  Fecal CgA Cell Density

 Granins represent crucial modulators of the intestinal microenvironment, impacting both the composition of luminal microbiota present in fecal samples and the adherent bacterial communities residing on the mucosal surface of the colon.^15,27^ Moreover, granins offer a two-pronged approach to controlling bacterial populations: their inherent acidity suppresses bacterial growth within the gut, while peptides derived from granins directly eliminate certain strains of bacteria.^28^ This dual action helps maintain a balanced gut microbiota, potentially alleviating IBS symptoms. However, further analysis revealed no correlation between granin profile clusters (fecal- or mucosal-dominant) and IBS symptoms, suggesting granin expression profiles alone may not predict the symptom severity.^24^ Additionally, examining different IBS subtypes (diarrhea-predominant, constipation-predominant, and neither) also showed no distinct patterns in protein levels or gene expression of specific granins across these groups.^15^

###  Duodenum and Ileum CgA Cell Density

 In the duodenum, a notable variance in CgA densities exists between all types of IBS and control groups (SDM = -12.08, 95% CI: -21.65, -2.51, *P* < 0.00001). However, within the IBS subgroup analysis, neither the IBS-C nor the IBS-D subgroup demonstrated a statistically significant association with the control group.

 Duodenum harbors the greatest diversity of gut endocrine cell types, including serotonin, secretin, cholecystokinin (CCK), gastric inhibitory polypeptide (GIP), somatostatin, and motilin.^9^ In cases of IBS, multiple studies have reported decreased densities of secretin, CCK, GIP, and somatostatin cells.^10^ Consequently, this reduction in those mentioned cell densities contributes to a decrease in CgA density in the duodenum.

 The analysis findings on the ileum indicated no statistically significant disparity between the IBS and control group (SMD = -0.84, 95% CI: -2.43, 0.76, *P* = 0.30), as revealed by outcomes from two quantitative investigations. The enteroendocrine cells found in the terminal ileum are peptide YY (PYY), pancreatic polypeptide (PP), enteroglucagon cells, and serotonin.^7,21^ A study hypothesized that the observed decrease in ileal endocrine cell density among the current patients with IBS could be attributed to reductions in Msi-1 and NEUROG3 cells. The diminished density of CgA cells could also potentially result from reductions in serotonin cell density, where serotonin plays a pivotal role in stimulating motility in the small and large intestines.^21^

###  Colon and Rectum CgA Cell Density

 In the colon, quantitative synthesis performed in two studies^8,16^ involving all types of patients with IBS showed significant differences in CgA cell densities in the right and left colon compared with the controls. Despite these findings, no significant difference was found in all types of IBS and IBS subgroups (IBS-C/IBS-D) compared with controls in all parts of the colon. However, the quantitative synthesis of CgA cell density in the colon showed trends toward IBS, suggesting a reduction in the density of total endocrine cells.

 In the rectum, the densities of CgA in IBS-total, IBS-D, IBS-M, and IBS-C did not differ from that in controls. Although CgA cell densities remained unaltered in patients with IBS, changes in specific endocrine cells should not be ruled out, as serotonin cells change in the rectum of patients with IBS.^29^ The different results of the colon and rectum can be explained by the physiological differences where the rectum’s only purpose is to preserve the feces before it is expelled, while the colon absorbs the water, salt, and several fat-soluble vitamins.^12^

 The current findings may offer a histopathological examination for IBS diagnosis from colonic biopsy, as the decrease in the CgA cell density would confirm the diagnosis of IBS. However, compared with colonoscopy, gastroscopy with duodenal biopsies of CgA cell density is preferred as it is quicker, simpler, and more comfortable for patients, while the colonic biopsy necessitates a total colonoscopy, which would be more complicated.^9^

###  Heterogeneity and Publication Bias Analysis

 Our analysis revealed significant heterogeneity in CgA cell density across various gastrointestinal segments, as evidenced by high I^2^ values for gastric antrum, corpus, ileum, duodenum, and all colonic regions (I^2^ = 98%, 99%, 92%, 99%, and 88%, respectively). Notably, the left and right colons displayed comparatively lower heterogeneity, with I^2^ values of 74% and 62%, respectively. This variability could stem from clinical, methodological, or statistical perspectives.

 From a clinical perspective, the differences in participants or outcomes could lead to high heterogeneity. The number of participants ranged from 11 to 219, with most studies having unequivocal male-to-female ratios and predominantly female subjects. The larger the sample, the higher the effect size likelihood. From a methodological perspective, the differences in study design may contribute to high heterogeneity. While the majority of studies employed the Rome III criteria for diagnosing IBS, two studies utilized the older Rome II criteria: Öhman et al^23^ and Sidhu et al,^13^ potentially introducing heterogeneity in the IBS diagnosis, nevertheless, both were not computed in the meta-analysis. Additionally, from a statistical perspective, the conversion from median to mean could lead to the imprecise CgA cell density in each outcome. However, it only occurred in a small number of studies.

 Potential bias sources in this study include where authors may intend to release papers without significant results. In this systematic review, each study showed various significance in their reported outcomes, as described in Table 2, reducing the likelihood of bias. Furthermore, funnel plot analysis of CgA concentration in the duodenum and colon revealed a relatively symmetrical distribution of studies around the central line, suggesting an absence of substantial publication bias.

## Strengths and Limitations of Study

 As far as our understanding extends, the current study marks the inaugural endeavor to conduct a systematic review and meta-analysis of CgA levels in patients with IBS. Our investigation covers the primary sites of CgA distribution within the GI tract, including the stomach, duodenum, ileum, and colon, thus providing a comprehensive representation of each segment. However, other areas of the GI tract have not been explored, highlighting the need for further research. Other limitations of this study include the inability to analyze the diagnostic accuracy of CgA due to the scarcity of available data. Furthermore, future research would benefit from a multicenter approach involving diverse countries worldwide rather than being limited to European nations.

###  Future Directions

 While CgA provides insights into overall endocrine activity, its lack of specificity limits its ability to accurately assess the severity of IBS symptoms or monitor disease progression. However, its potential to reflect general hormonal changes may be valuable in understanding broader trends or responses to interventions that affect endocrine function in patients with IBS. Further research, including direct comparisons of CgA sources, is necessary to determine which sources are most reliable and effective for diagnosing IBS. Additionally, future studies with larger and more diverse samples are needed to enhance the generalizability of the findings and strengthen the implications of the results.

## Conclusion

 Overall, the quantitative synthesis of CgA cell density showed trends toward IBS compared with control groups. Significant CgA concentration differences were found in the left colon, right colon, and duodenum. Therefore, current findings might offer a histopathological approach to confirm the IBS diagnosis.

## Supplementary Files

Supplementary file contains Tables S1-S6.
