# Supplementary material for: Chromogranin A as a Novel Biomarker of Irritable Bowel Syndrome in Adults: A Systematic Review and Meta-Analysis
Source: Middle East J Dig Dis. 2025 Apr 30;17(2):128–38. doi: 10.34172/mejdd.2025.418 (PMC12456167; doi:10.34172/mejdd.2025.418)
Supplement: Supplementary file 1 — Supplementary file contains Tables S1-S6. [file mejdd-17-128-s001.pdf]

Middle East J Dig Dis. April 2025;17(2):128-S1
10.34172/mejdd.2025.418
http://mejdd.org

Supplementary File 1.

Table S1. PECOTS-SD

Patients	Adult patients (≥18 years) with a primary diagnosis of IBS confirmed by Rome criteria, a smoke-free lifestyle, and no gastrointestinal medications within the past 48 hours or more.
Exposure	All types of IBS include IBS related constipation (IBS-C), IBS related diarrhea (IBS-D), or IBS mixed (IBS-M)
Comparator	Healthy patients (control)
Outcomes	Chromogranin A (CgA) cell density
Time	No publication’s time restriction
Setting	Participants visiting medical facility
Study Design	Observational study design

PECOTS-SD (participant, exposure, comparator, outcomes, time, setting, study design)

Table S2. Search Terms and Strategy: **PubMed/MEDLINE**

Search Number	Query	Filter	Results
1	("Chromogranins"[Mesh]) OR "Chromogranin A"[Mesh]	Clinical Trial, Observational Study, Full Text	134

2	"Irritable Bowel Syndrome"[Mesh]	Clinical Trial, Observational Study, Full Text	1,052
3	("Diarrhea"[Mesh]) OR "Constipation"[Mesh]	Clinical Trial, Observational Study, Full Text	5,676
4	#1 AND (#2 OR #3)	Clinical Trial, Observational Study, Full Text	22

Table S3. Search Terms and Strategy: **ProQuest**

Search Number	Query	Filter	Results
1	("Chromogranins" OR "Chromogranin A" OR "secretogranin")	Scholarly Journals, Full text, Document type: Article, Subject: humans	1,536
2	"Irritable Bowel Syndrome"	Scholarly Journals, Full text, Document type: Article, Subject: humans	3,817
3	("Diarrhea" OR "Constipation")	Scholarly Journals, Full text, Document type: Article, Subject: humans	32,995
4	#1 AND (#2 OR #3)	Scholarly Journals, Full text, Document type: Article, Subject: humans	465

Table S4. Search Terms and Strategy: **Science Direct**

Search Number	Query	Filter	Results
1	("Chromogranins" OR "Chromogranin A" OR "secretogranin")	Research articles, Open access and Open archive, Medicine and Dentistry	1,130

2	"Irritable Bowel Syndrome"	Research articles, Open access and Open archive, Medicine and Dentistry	1,746
3	("Diarrhea" OR "Constipation")	Research articles, Open access and Open archive, Medicine and Dentistry	27,942
4	#1 AND (#2 OR #3)	Research articles, Open access and Open archive, Medicine and Dentistry	361

Table S5. Search Terms and Strategy: **EBSCOHost**

Search Number	Query	Filter	Results
1	("Chromogranins" OR "Chromogranin A" OR "secretogranin")	Online Full text, scholarly peer-reviewed journals	987
2	"Irritable Bowel Syndrome"	Online Full text, scholarly peer-reviewed journals	1,119
3	("Diarrhea" OR "Constipation")	Online Full text, scholarly peer-reviewed journals	18,975
4	#1 AND (#2 OR #3)	Online Full text, scholarly peer-reviewed journals	375

Table S6. Characteristics of the included studies

Author, Publication Year, Country	Study Designs	Groups: IBS group Age (y)	IBS group Sex N (%)	Control group Age (y)	Control group Sex N (%)	Population characteristics	Inclusion/ Exclusion Criteria of Participants	Chromogranin detection method	Chromogranin Sources	IBS Types	Summary of the findings
El-Salhy et al[9] 2010, Norway	Case-control study	35 (range 18–58)	Female: 39 (95.1%) Male: 2 (4.9%)	41 (range 18 - 63)	Female: 37 (62.7%) Male: 22 (37.3%)	IBS group: A total of 41 patients with IBS symptoms (diarrhea, constipation, or both) fulfilled the Rome Criteria III. Control group: A total of 59 healthy patients comprised volunteers, medical students, and hospital employees, without any complaints.	IBS group comprised of patients with symptoms that fulfilled Rome III Criteria	Chromogranin plasma concentration was measured using the ELISA technique using an ALPCO Diagnostics kit.	• Duodenum • All parts of the colon • Right colon • Left colon	• 23 patients had diarrhea (IBS-diarrhea/ IBS-D). • 18 patients had constipation (IBS-constipation/IBS-C).	• There was no statistically significant difference in CgA plasma concentrations between controls and IBS patients (*P*=0.4). • These findings were also true for both IBS subtypes (*P*=0.4 and 0.5, respectively).
El-Salhy et al[12] 2012, Norway	Case-control study	38 (range 18 - 65)	Female: 39 (4.9%) Male: 8 (95.1%)	49 (range 18 - 67)	Female: 19 (62.7%) Male: 8 (37.3%)	IBS group: A total of 47 patients with IBS that fulfilled the Rome Criteria III. Control group: A total of 27 healthy subjects without any gastrointestinal complaints.	IBS group comprised patients with symptoms that fulfilled Rome III Criteria	The main antibody utilized was a mouse monoclonal anti-N-terminal purified CgA antibody.	Rectum, approximately 15 cm from the anus	• 28 patients with IBS-D. • 19 patients with IBS-C.	There was no statistically significant difference between the controls, IBS, IBS-D, or IBS-C patients (*P*=0.5, 0.5, and 0.7, respectively).
El-Salhy et al[7] 2013, Norway	Case-control study	35 (range 18 - 66)	Female: 77 (78.6%) Male: 21 (21.4%)	52 (range 20 - 69)	Female: 16 (59.3%) Male: 11 (40.7%)	IBS group: A total of 98 patients with IBS, according to the Rome III Criteria Control group: A total of 27 healthy subjects without any gastrointestinal complaints.	IBS group comprised patients with symptoms persisted for years, and not associated with any event (gastrointestinal or other infections).	Monoclonal mouse anti-N-terminal purified CgA was employed as the primary antibody.	Ileum, cecum, ascending colon, right and left part of transverse colon, descending colon, and sigmoid colon.	• 35 patients with IBS-D. • 32 with IBS-C. • 31 patients with IBS-M (mixture of diarrhea and constipation)	The difference between controls and all IBS patients (IBS-D, IBS-M, and IBS-C; P0.0001 for all) was statistically significant.
El-Salhy et al[20] 2014a, Norway	Case-control study	36 (range 18 – 66)	Female: 180 (88.7%) Male: 23 (11.3%)	38 (range 18 – 67)	Female: 77 (89.5%) Male: 9 (10.5%)	IBS group: A total of 203 patients with IBS who fulfilled Rome III criteria referred to Stord Hospital during 2002–2011. Control group: A total of 86 healthy subjects, without any gastrointestinal complaints, recruited from Stord Hospital, Haukelands University Hospital, and the University of Bergen.	IBS group comprised patients with symptoms persisted for many years and could not associate the onset of IBS symptoms with any event, particularly gastrointestinal or other infections.	A monoclonal mouse antibody produced against the N-terminus of pure CgA was employed as the main antibody.	Duodenum	• 80 patients with IBS-D. • 76 patients with IBS-C. • 47 patients with IBS-M.	• The number of CgA cells in IBS-total, IBS-D, IBSSM, and IBS-C patients was considerably lower than controls (*P*<0.0001 for all types). • CgA cell density was lower in IBS-total and all IBS-subgroups than in controls (*P*<0.0001).
El-Salhy et al[6] 2014b, Norway	Case-control study	32 (range 18 - 55)	Female: 62 (81.6%) Male: 14 (18.4%)	38 (range 20 - 67)	Female: 43 (72.9%) Male: 16 (27.1%)	IBS group: A total of 76 patients fulfilled Rome III Criteria for IBS. Control group: A total of 59 healthy subjects, 15 from the population of Stord City and 44 were students or hospital employees, recruited at Stord	IBS group comprised patients with symptoms persisted for many years and could not associate the onset of IBS symptoms with any event,	A monoclonal mouse antibody produced against the N terminus of pure CgA was employed as the main antibody.	Gaster (stomach), the antra and corpora parts	• 26 patients with IBS-D. • 29 patients with IBS-C. • 21 patients with IBS-M.	• CgA cell density varied substantially between controls, IBS total, and IBS subgroups (*P*=0.01). • In comparison to the controls, CgA cell density was considerably lower in the IBSM group and significantly greater in the IBSC group (*P*=0.003 and *P*=0.01, respectively.

Author, Publication Year, Country	Study Designs	Groups				Population characteristics	Inclusion/ Exclusion Criteria of Participants	Chromogranin detection method	Chromogranin Sources	IBS Types	Summary of the findings
		IBS group		Control group							
		Age (y)	Sex N (%)	Age (y)	Sex N (%)						
						Hospital, Haukelands University Hospital, and the University of Bergen.	particularly gastrointestinal or other infections.				• AntiCgA immunoreactivity levels were 122.21.4, 126.00.8, 125.70.9, 124.73.7, and 127.01.4 in controls and IBS total, IBS-D, IBS-M, and IBS-C patients, respectively.
El-Salhy et al[21] 2017, Thailand and Norway	Case-control study	Thai subjects: 56 (range 45–70)<br><br>Norwegian subjects: 49.2 (range 29–70)	Thai subjects: Female: 23 (76.7%) Male: 7 (23.3%)<br><br>Norwegian subjects: Female: 40 (85.1%) Male: 7 (14.9%)	Thai subjects: 50 (range 36 – 69)<br><br>Norwegian subjects: 50.3 (range 38–67)	Thai subjects: Female: 14 (70%) Male: 6 (30%)<br><br>Norwegian subjects: Female: 15 (75%) Male: 5 (25%)	Thai subjects:<br>IBS group:<br>Thirty patients who fulfilled the Rome III criteria for the diagnosis of IBS were recruited at King Chulalongkorn Memorial Hospital, Bangkok, Thailand.<br><br>Norwegian subjects:<br>IBS group:<br>47 patients with IBS according to the Rome III criteria were recruited at Stord Hospital, Stord, Norway.	• IBS group comprised patients with symptoms persisted for many years and could not associate the onset of IBS symptoms with any event, particularly gastrointestinal or other infections.<br><br>• Patients who had used antibiotics, immunosuppressants, or nonsteroid anti-inflammatory drugs within one month of trial participation were not eligible.	The main antibody utilized was a 1:1000 dilution of a monoclonal mouse antibody produced against the N-terminus of pure CgA.	Sigmoid colon and rectum	Thai subjects:<br>• 11 patients with IBS-D.<br>• 13 patients with IBS-C.<br>• A total of six patients with IBS-M.<br><br>Norwegian subjects:<br>• 18 patients with IBS-D.<br>• 15 patients with IBS-C.<br>• 14 patients with IBS-M.	Colon samples:<br>• No statistically significant differences in CgA cell density between controls and people with IBS-total, IBS-D, IBS-M, or IBS-C (*P*=0.3, *P*=0.9, *P*=0.7, and *P*=0.07, respectively).<br>• A significant difference in the Norwegian participants between controls, IBS-total, and IBS subgroups (p=0.007).<br>• CgA cell density was lower in IBS overall, IBS-D, IBS-M, and IBS-C patients than in controls (*P*=0001, *P*=0.002, *P*=0.005, and *P*=0.0007, respectively).<br>Rectum samples:<br>• The density of CgA did not differ between Thai controls, total-IBS, and the IBS subgroups (*P*=0.9).<br>• The densities of CgA in IBS-total, IBS-D, IBS-M, and IBS-C did not differ from that in controls (*P*=0.9, *P*=0.6, *P*=0.8, and *P*=0.7, respectively).<br>• No significant difference in the CgA cell density between Norwegian controls, IBS-total, and the IBS subgroups (*P*=0.7).
Mujagic et al[14] 2016, Netherlands	Case-control study	Mean age 44.8 ± 16.4 (range 18–75)	Female: 138 (70.4%) Male: 58 (29.6%)	Mean age 43.9 ± 19.2 (aged >18)	Female: 98 (61.3%) Male: 62 (38.7%)	IBS group:<br>Those who fulfilled Rome III criteria via the outpatient Gastroenterology-Hepatology Clinic of Maastricht University Medical Center+ (MUMC+)<br>Control group:<br>Randomly enrolled from a large general population cohort in the Netherlands	Patient with a history of abdominal surgery, apart from appendectomy, laparoscopic cholecystectomy, and hysterectomy	CgA was detected using a commercial radioimmunoassay (RIA, Euro-Diagnostica, Sweden)	Fecal; not specified	• 71 patients with IBS-D.<br>• 34 patients with IBS-C.<br>• 78 patients with IBS-M.<br>• 13 patients with IBS-U.	• CgA (*P*=0.001) and fecal calprotectin (*P*<0.001) were significantly increased in the IBS group compared to other biomarkers.<br>• Markers with a negative regression coefficient are increased in the IBS group (IL-12, TNF-a, CgA, calprotectin)
Mujagic et al[22] 2017, Netherlands	Case control study	IBS Hypersensitive	IBS Hypersensitive	IBS Normosensitive	IBS Normosensitive	IBS group:<br>Those who fulfilled Rome III criteria via the outpatient Gastroenterology-	Patient with a history of abdominal surgery,	CgA was detected using a commercial radioimmunoassay	Fecal; not specified	**IBS hypersensitive** (D/C/M/U): 26.5 /	CgA was found lower in the IBS Hypersensitive group than IBS Normosensitive group (22.04 ± 26.86 vs

Author, Publication Year, Country	Study Designs	Groups				Population characteristics	Inclusion/ Exclusion Criteria of Participants	Chromogranin detection method	Chromogranin Sources	IBS Types	Summary of the findings
		IBS group		Control group							
		Age (y)	Sex N (%)	Age (y)	Sex N (%)						
		Mean age 37.7 ± 15.8	Female: 68 (81.9%) Male: 15 (18.1%)	Mean age 46.7 ± 16.3	Female: 58 (69%) Male: 26 (31%)	Hepatology Clinic of Maastricht University Medical Center+ (MUMC+)	apart from appendectomy, laparoscopic cholecystectomy, and hysterectomy	(RIA, Euro-Diagnostica, Sweden)		24.1 / 42.2 / 7.2 **IBS Normosensitive** (D/C/M/U): 35.7 / 14.3 / 40.5 / 9.5	24.8 ± 29.8), but there was no significant difference between those groups
Öhman et al[23] 2012, Sweden	Case-control study	38 (range 19–68)	Female: 59 (72%) Male: 23 (28%)	38 (range 23–63)	Female: 22 (57.8%) Male: 16 (42.2%)	IBS group:<br>Patients were recruited from an outpatient clinic at Sahlgrenska University Hospital Gothenburg, Sweden.<br>IBS patients were subgrouped according to the predominant bowel habit (Rome II criteria)<br>Control group:<br>Healthy volunteers with no history of gastrointestinal disorders and bowel-related symptoms during the last 7 days	Patients with a history of taking any medications that affect the gastrointestinal tract or the immune system	CgA and CgB were collected from fecal samples and measured with commercial radioimmunoassays (Eurodiagnostica, Malmö, Sweden)	Fecal; not specified	• 36 patients with IBS-D.<br>• 15 patients with IBS-C.<br>• 31 patients with IBS-A.<br>*A = alternating	• Fecal CgA was higher in IBS patients compared to healthy patients (*P* 0.009).<br>• All types of IBS demonstrated higher levels of CgA compared to control, but only IBS-D was statistically significant (*P*<0.01).<br>• CgA (AUC=0.66) was not useful for discriminating IBS patients from healthy patients.<br>• Fecal CgA level was associated with the average stool frequency and abdominal pain but not with colonic transit time.
Pletikosic et al[24] 2015, Croatia	Cross sectional study	45.2 ± 14.15	Female: 36 (77%) Male: 11 (23%)	-	-	Not mentioned	Not mentioned	Not mentioned	Serum	Not mentioned	• CgA was absent in nearly 46% of the sampled cases, and 29% of sample data pertaining to CgA levels was unavailable.<br>• Within the remaining subset of the sample (n=12), CgA concentrations varied between 6 and 163 u/L, and four patients exhibited CgA levels exceeding 60 u/L.
Mazzawi et al[8] 2016, Norway	Prospective cohort study	33 (range 24–44)	Female: 7 (63.6%) Male: 4 (36.4%)	54 (range 26–70)	Female: 9 (64.3%) Male: 5 (35.7%)	IBS group:<br>Patients of both genders were referred to the Division of Gastroenterology, Stord Hospital, Stord, Norway, and fulfilled Rome-III criteria for IBS.<br>Patients were instructed not to consume any proton pump inhibitors and antacids one week prior the study.	The IBS group comprised patients with symptoms that fulfilled Rome III Criteria.	The primary monoclonal mouse antibody raised against the N-terminal of purified CgA was diluted to 1:1000.	Duodenum, Ileum	Not mentioned	• CgA-immunoreactive cells were identified in the mucosal linings of both the duodenum and ileum, predominantly localized within the crypts, in individuals diagnosed with IBS and in control subjects.<br>• CgA-immunoreactive cells in the duodenum in the patients with IBS was lower than in control (*P*<0.0001).<br>• CgA-immunoreactive cells in the ileum in the patients with IBS was slightly higher than in control (*P* =0.99).
Mazzawi et al[10] 2015, Norway	Prospective cohort study	34 (range 20-45)	Female: 8 (61.5%) Male: 5 (38.5%)	54 (range, 20-70)	Female: 9 (64.3%) Male: 5 (35.7%)	IBS group:<br>Individuals with IBS who met the Rome-III criteria and were sent to the gastroenterology division at Stord Hospital in Norway ranged in age from 18 to 70.	IBS patients, according to the Rome-III criteria, aged 18–70 years, with no organic gastrointestinal or	Two hours of incubation with a monoclonal mouse anti-N-terminal of purified CgA primary antibody	Colon, rectum	Not mentioned	• There was a significant increase in the densities of CgA cells in the total colon before and after receiving dietary guidance (*P* = 0.0004).<br>• The increases in CgA cell densities in both the left colon and right colon were

Author, Publication Year, Country	Study Designs	Groups				Population characteristics	Inclusion/ Exclusion Criteria of Participants	Chromogranin detection method	Chromogranin Sources	IBS Types	Summary of the findings
		IBS group		Control group							
		Age (y)	Sex N (%)	Age (y)	Sex N (%)						
						Control group:<br>Healthy subjects that underwent gastroscopy due to gastrointestinal bleeding reason and health concerns due to gastrointestinal cancer diagnosis in family member	any other systemic diseases, drug abuse, or psychiatric illness, history of abdominal surgery.	(code number M869; Dako, Glostrup, Denmark) diluted to 1:1000 at room temperature.			also statistically significant (*P* = 0.0157 and 0.0039, respectively).<br>• There was no significant difference in the cell density in the rectum between pre- and post-dietary guidance (*P* = 0.47).
Mazzawi et al[11] 2014, Norway	Prospective cohort study	34 (range 20-45)	Female: 9 (64.3%)<br>Male: 5 (35.7%)	54 (range, 20-70)	Female: 9 (64.3%)<br>Male: 5 (35.7%)	IBS group:<br>Rome-III IBS criteria were fulfilled for patients who were referred to a clinic at Stord Helse-Fonna Hospital (Stord, Norway)<br>Control group:<br>Healthy subjects that underwent gastroscopy due to gastrointestinal bleeding reason and health concerns due to gastrointestinal cancer diagnosis in family member	IBS patients fulfilled the Rome-III criteria with no organic gastrointestinal or any other systemic diseases, drugs abused, or psychiatric illness, history of abdominal surgery.	The IBS patients' corpus and antrum were assessed for CgA density using monoclonal mouse anti-N-terminal of purified CgA primary antibody (code M869; Dako, Glostrup, Denmark) diluted 1:1000.	Corpus, antrum of gaster	Not mentioned	• A significant increase in CgA-secreting cell densities in the corpus of IBS patients after dietary guidance (*P*=0.0064).<br>• Before and after dietary guidance, no significant difference in the densities of CgA-secreting cells in antrum was detected in IBS patients (*P*=0.2).
Sidhu et al[13] 2009, United Kingdom	Prospective cohort study	45 (17-88)	Female: 150 (68.5%)<br>Male: 69 (31.5%)	N/A	N/A	IBS:<br>Patients who met the Rome II criteria for D-IBS were selected from the outpatient department of Royal Hallamshire Hospital in Sheffield, United Kingdom.<br><br>Control:<br>No healthy control comparison	Patients who fulfilled the Rome II criteria of D-IBS	Purified full-length human CgA was used in a competitive radioimmunoassay to measure serial CgA levels.	Serum	IBS-D	• In IBS-D patients, CgA levels appeared to be transiently elevated.<br>• Normal CgA levels (0-20u/l) were found in 81% of IBS patients (n=177), whereas 12.3% (n=27) had values between 20-60u/l, 6.8% (n=15) had CgA levels >60u/l.<br>• 96% of patients with baseline CgA levels of 20-60u/l had repeated CgA levels that returned to normal following the resolution of symptoms while receiving standard therapy.
